# Isolation and characterization of *Treponema phagedenis*-like spirochetes from digital dermatitis lesions in Swedish dairy cattle

**DOI:** 10.1186/1751-0147-50-40

**Published:** 2008-10-20

**Authors:** Märit Pringle, Christer Bergsten, Lise-Lotte Fernström, Helena Höök, Karl-Erik Johansson

**Affiliations:** 1 Dept of Biomedical Sciences and Veterinary Public Health, Swedish University of Agricultural Sciences, Box 7009, SE-75007 Uppsala, Sweden; 2 Dept of Animal Environment and Health, Swedish University of Agricultural Sciences, Box 234, SE-53223 Skara, Sweden

## Abstract

**Background:**

Digital dermatitis in cattle is an emerging infectious disease. Ulcerative lesions are typically located on the plantar skin between the heel bulbs and adjacent to the coronet. Spirochetes of the genus *Treponema *are found in high numbers in the lesions and are likely to be involved in the pathogenesis. The aim of this study was to obtain pure cultures of spirochetes from cattle with digital dermatitis and to describe them further.

**Methods:**

Tissue samples and swabs from active digital dermatitis lesions were used for culturing. Pure isolates were subjected to, molecular typing through 16S rRNA gene sequencing, pulsed-field gel electrophoresis (PFGE), random amplified polymorphic DNA (RAPD) and an intergenic spacer PCR developed for *Treponema *spp. as well as API-ZYM and antimicrobial susceptibility tests. The antimicrobial agents used were tiamulin, valnemulin, tylosin, aivlosin, lincomycin and doxycycline.

**Results:**

Seven spirochete isolates from five herds were obtained. Both 16S rRNA gene sequences, which were identical except for three polymorphic nucleotide positions, and the intergenic spacer PCR indicated that all isolates were of one yet unnamed species, most closely related to *Treponema phagedenis*. The enzymatic profile and antimicrobial susceptibility pattern were also similar for all isolates. However it was possible to separate the isolates through their PFGE and RAPD banding pattern.

**Conclusion:**

This is the first report on isolation of a *Treponema *sp. from cattle with digital dermatitis in Scandinavia. The phylotype isolated has previously been cultured from samples from cattle in the USA and the UK and is closely related to *T. phagedenis*. While very similar, the isolates in this study were possible to differentiate through PFGE and RAPD indicating that these methods are suitable for subtyping of this phylotype. No antimicrobial resistance could be detected among the tested isolates.

## Background

Digital dermatitis (DD) is a contagious claw disease causing lameness in cattle, most commonly seen in intensive dairy production. The disease was first described in 1974 in Italy by Cheli and Mortellaro [1]. In Sweden the first herd with DD was described recently [2] whereas previously only sporadic, atypical cases have been reported [3]. There is a strong connection between wet/dirty claw environments and the occurrence of DD [4], for example in cubicle systems where accumulation of faeces and urine on the alleys is a typical hygienic problem. Besides being an animal welfare problem, economic losses due to reduced milk production and weight loss are associated with DD [5].

The rapid response to antibiotic treatment of DD lesions strongly supports a bacterial cause. Many bacteria of different genera, such as *Treponema*, *Fusobacterium, Dichelobacter, Prevotella*, and *Porphyromonas *have been isolated from DD lesions and a polymicrobial cause is often discussed. However, there is strong circumstantial evidence that *Treponema *spp. are central in the aetiology of DD. As early as 1964 Gupta et al. observed spirochetes in smears from different variants of "foot-rot" manifestations in cattle [6]. Another early observation of spirochetes was made 1988 by Blowey and Sharp when DD was described for the first time in the UK [7]. The first spirochete cultures from DD were reported by Walker et. al 1995 [8]. In histological preparations from DD lesions treponemes are found invading the deeper layers of epidermis [9]. Additionally a humoral immune response against *Treponema *spp. has been demonstrated in infected cattle [10,11]. Successful experimental transmission of the disease through inoculation with fresh scrapings from DD lesions was described in 1996 [12]. It was also confirmed by histopathology that spirochetes invaded the tissue 1–2 weeks after inoculation [13].

Only about 40 isolates of spirochetes from DD have been described [8,11,14-16]. Through 16S rRNA gene sequencing all these isolates have been determined to be members of the genus *Treponema*. However, to date no successful experimental infection in cattle with bacterial cultures has been reported.

More than one phylotype of *Treponema *can be present in the same lesion. Different phylotypes have been isolated from the same animal [8,16] and by cloning and sequencing of 16S rRNA genes, five different phylotypes were identified in a pooled sample from four cows [17]. Several phylotypes in the same lesion have also been demonstrated by fluorescence in situ hybridization (FISH) on biopsies [9,18]. Some phylotypes have not yet been reported as cultured.

Standard methods often have to be adjusted to suit the treponemes. Some methods have shown promising results. For example Stamm et al. have developed a method for rapid phylotyping of *Treponema *spp. based on sequence length difference of the intergenic spacer between the genes for 16S and 23S rRNA [19]. A PCR for the intergenic spacer region 2 (ISR2) containing a tRNA^Ile ^gene gives products that vary sufficiently in size, for the difference to be visualized on an ordinary electrophoresis gel. For studies of clonal relationship pulsed-field gel electrophoresis (PFGE) has been used. Six *T. phagedenis*-like isolates, of which four were from the same herd, were shown to have unique PFGE patterns after cleavage with *Xba*I, *Not*I and *Sse*8387I [11].

In countries where DD is widespread, footbaths containing antibiotics are often used. These footbaths rapidly become contaminated with faeces and dirt and hence function as large selective cultures of antibiotic resistant bacteria. In Sweden tetracyclines are used, but only for topical treatment of individual animals since on herd level footbaths with copper sulphate are recommended. Except for natural rifampin resistance [20] there are no previous reports on antimicrobial susceptibility of the *Treponema *spp. from cattle with DD.

The objectives of this study were to obtain pure cultures of *Treponema *spp. from DD lesions in Swedish dairy cattle and to subject the cultures to genotypic and phenotypic characterization.

## Methods

### Bacterial isolates and growth conditions

The spirochete isolates were obtained by culture from clinical submissions of tissue samples transported in isotonic saline or swabs transported in Amies. medium with charcoal (COPAN, Brescia, Italy). All samples were from active DD lesions diagnosed by a veterinarian. The selective medium used was FABSA (fastidious anaerobe broth, LabM, with 25% fetal calf serum, National Veterinary Institute, Uppsala, Sweden, rifampicin Sigma, Sigma-Aldrich Sweden AB, 10 μg/ml and enrofloxacin Fluka, Sigma-Aldrich Sweden AB, 10 μg/ml). All cultures were grown on a shaker (90 rpm), in an anaerobic atmosphere, in jars. The purity of the cultures was checked by phase contrast microscopy. When pure cultures were obtained, non selective broth FABS (FABSA without antimicrobial agents), was used. The pure isolates were stored in FABS with 20% glycerol at -70°C.

### DNA sequencing and analysis

DNA was prepared from broth cultures. The cells were washed twice in PBS, boiled for 10 min in sterile water and cell debris removed by centrifugation. The primers used for amplification of the 16S rRNA gene were originally developed for the spirochete genus *Brachyspira *[21]. Sequencing with an ABI Prism 3100 analyser and the sequence analysis was performed as previously described [21].

### ISR2 PCR

The DNA was prepared as for the 16S rRNA gene amplification. A PCR protocol for amplification of the 16S-tRNA^Ile ^region of ISR2 developed by Stamm et al. was followed [19]. The sequence of the product from one isolate from the ISR2 PCR was determined using the PCR primers as sequencing primers.

### Antimicrobial susceptibility

The tests were made by broth dilution in a panel designed for susceptibility tests of *Brachyspira *spp. (VetMIC™ brachy QCR low, National Veterinary Institute, Uppsala, Sweden) [22]. The panel included tiamulin, valnemulin, tylosin, aivlosin, lincomycin and doxycycline, dried in tissue culture trays with 48 wells (Nunclon™Δ Multidishes, NUNC™, Denmark) in twofold serial dilutions. The possible influence of the high serum content in the FABS broth and the exceptionally long incubation time was tested with *Staphylococcus aureus *(ATCC 29213) and *Brachyspira hyodysenteriae *B78^T ^(ATCC 271 64^T^) under identical conditions as for the *Treponema *isolates. From a fully grown *Treponema *broth culture (5 ± 1 days old) 300 μl was transferred to 30 ml FABS broth. Each well in the panels was filled with 0.5 ml of the inoculum. The panels were incubated in square GENbox anaerobic jars (bioMérieux, Lyon, France) with AnaeroGen generator sachets (Oxoid, Hampshire, UK). The panels were covered with plastic lids, with a maximum of four panels per jar, and incubated on a rotary shaker (90 rpm) at 37°C. When the growth in the wells was sufficient for reading the endpoints (varied between 5 to 11 days) the MIC was read as the lowest concentration of the antimicrobial agent that prevented visible growth. One well in each panel containing no drug served as growth control, and was used for visual comparison with growth in the other wells. The reading was made with the assistance of a viewing device with a mirror, to obtain indirect light.

### API-ZYM

Bacterial cells from 5–6 days old broth cultures (FABS) were washed twice in isotonic saline. The cells were suspended in isotonic saline to a density of 5–6 McFarland. The API-ZYM test (bioMérieux) was performed following the manufacturers instruction. The strips were incubated aerobically for 4 h at 37°C. To test the influence of buffer and incubation atmosphere, one isolate (V1) was suspended in both isotonic saline (pH 6.3) and phosphate buffered saline (PBS, pH 7.3) and incubated both aerobically and anaerobically. As control strain the recommended *Pseudomonas aeruginosa *(ATCC 27853) was used.

### Pulsed-field gel electrophoresis

The PFGE protocol was modified from a protocol for *Campylobacter *spp. and the restriction enzyme was chosen from the study performed by Trott et al. [11,23]. Bacterial cells from a 15 ml broth (FABS) culture were washed three times in TE buffer (10 mM Tris, 1 mM EDTA). The bacterial pellet was resuspended in 1–1.5 ml Pett IV buffer (10 mM Tris-HCl, 1 M NaCl), to obtain an optical density of 2.0–2.5 at 405 nm, and mixed with an equal amount of 1% low melting temperature agarose (InCert^® ^Agarose, Cambrex Bio Science Rockland, Inc., Maine, USA). Gel plugs were incubated in ESP (0.5 M EDTA, 1% N-Lauroyl sarcosine, 0.2% Pronase E) at 50°C for 24 h, with refreshing of the solution after 1.5 h. Before restriction digestion the gel plugs were washed in TE buffer (10 mM Tris, 1 mM EDTA) six times. Plug slices of 1–2 mm were digested with 30 units of *Xba*I in the recommended buffer (Amersham Biosciences, Buckinghamshire, UK) for 16 h at 37°C on a shaker. Lambda Ladder PFG Marker N0340S (New England BioLabs, Inc., Massachusetts, USA) was used as a molecular size marker. The restriction fragments were separated in a 0.9% agarose gel (SeaKem^® ^Gold Agarose, Cambrex Bio Science) using the CHEF-DR^® ^III Pulsed Field Electrophoresis System (Bio-Rad Laboratories AB, Sundbyberg, Sweden). The PFGE was performed in 0.5× TBE buffer (44.5 mM Tris, 44.5 mM boric acid, 1 mM EDTA) at 6 V/cm^2 ^and 14°C with a 120° including angle for the pulsed field. The initial switch time was 1.2 s, the final switch time 54 s and the gel was run for 19 h. The gels were stained with ethidium bromide and visualised using a GelDoc™ XR gel documentation system (Bio-Rad Laboratories AB).

### Random amplified polymorphic DNA

The DNA was prepared as for the 16S rRNA gene amplification. The RAPD reactions were performed in a Perkin-Elmer GeneAmp^® ^PCR System 2400 thermal cycler (Applied Biosystems, Stockholm, Sweden) at 100% ramp rate and a Ready-To-Go™ RAPD kit (GE Healthcare, Uppsala, Sweden) was used. The kit contains six different primers of which only Primer 4 (5'-AAGAGCCCGT-3') gave discriminatory banding pattern for the *Treponema *sp. in this study. The protocol in the kit was followed except for the gel electrophoresis that was performed in a 1.5% gel (SeaKem^® ^LE Agarose, Cambrex Bio Science) for 30 min at 80 V followed by 45 min at 100 V.

### Nucleotide sequence accession numbers

The nucleotide sequences of the 16S rRNA gene and ISR2 fragment were deposited in GenBank under accession numbers DQ470655, DQ470656, EF057411, EU375741 – EU375744 and EU410484.

## Results

### Isolation of spirochetes

Pure spirochete cultures were obtained from five different farms. Five isolates were from swabs, one from a biopsy and one from tissue collected at slaughter. For the origin of the isolates see Table 1. All isolates had a uniform morphology and the motility appeared to be more vigourous at the cell ends, as judged by phase contrast microscopy. Because of slow and confluent growth it was not possible to pick single colonies subcultured on agar. However, raw sequence data without any traces of contamination, using non species specific *Treponema *primers both for the 16S rRNA gene and the intergenic spacer, indicate that > 95% of the cultures were of one species. Additionally when different DNA preparations from the same isolate were analysed by PFGE and RAPD, identical banding patterns also indicate that the cultures contained a single species.

**Table 1:** Origin of the *Treponema *sp. isolates from Swedish cattle.

Isolate	Animal origin	County code	Year of isolation	Comment
V1	herd A	O	2005	about 20% clinical DD
T 413	herd B	C	2006	sporadic cases of DD in the herd
T 551	challenge study^a^	O	2006	isolated 25 days post infection
T 551B	challenge study^a^	O	2006	isolated 41 days post infection
T 603	herd C	C	2006	sporadic cases of DD in the herd
T 657	herd C	C	2006	sporadic cases of DD in the herd
T 2378	herd D	O	2005	sporadic cases of DD in the herd

^a ^unpublished, see discussion.

### 16S rRNA gene analysis

All isolates had identical 16S rRNA sequence except for two isolates (T 603 and T 657) that had three polymorphic nucleotide positions 133_Y_, 794_R _and 1138_Y _(*Escherichia coli *numbering). These three positions were polymorphic with two nucleotides in 50% representation and all were present in sequences from both strands. The 16S rRNA gene sequence was also identical to deposited sequences from DD treponemes isolated in California, Iowa, and the UK [8,11,16]. The most closely related treponeme was *T. phagedenis*.

### ISR2 PCR

A single band with a size of slightly more than 300 bp was recorded for all seven isolates. The length of the sequenced product from isolate V1 was 280 bp when the primer sequences were removed. Compared to available sequences in GenBank, 172 of 172 nucleotides including the intergenic spacer between 16S rRNA and the tRNA^Ile ^genes, which is the most variable region of the sequenced fragment, was identical to that of a Californian isolate, 2-1498 [GenBank: AF179261] [19].

### Antimicrobial susceptibility

The MICs of six antimicrobial substances for the *Treponema *sp. isolates and the control strains are presented in Table 2, 3, 4. The control strain tests were within proposed or accepted ranges except for tiamulin and valnemulin MICs that were one twofold dilution above the range for *Brachyspira hyodysenteriae *B78^T ^(ATCC 27164^T^) (Table 2). On repeated tests of isolate V1 the MICs only varied one twofold dilution step (Table 3). The results for the seven *Treponema *sp. isolates were very similar and no high MICs of the antimicrobial agents included were recorded (Table 4).

**Table 2:** MIC of six antimicrobial substances for two control strains from two tests under identical conditions as for the *Treponema *sp. isolates.

Strain	Days of incubation	MIC (μg/ml)					
		
		Tiamulin	Valnemulin	Tylosin	Aivlosin	Lincomycin	Doxycycline
*Staphylococcus aureus * CCUG 15915	4 and 7	>1 0.5–2^a^	0.25–0.5	2 0.5–4^a^	4	1–2	0.5 0.12–0.5^a^
							
*Brachyspira hyodysenteriae* B78^T ^ATCC 27164^T^	7 and 11	0.125 0.016–0.063^b^	0.063 0.008–0.031^b^	8–16 2–16^b^	1–2 0.5–4^b^	0.5–1 0.125–1^b^	0.25 0.063–4^b^

^a ^Approved quality control ranges by the CLSI for susceptibility tests performed by micro dilution in Mueller Hinton broth [25,26].

^b ^Proposed quality-control ranges by Pringle et al. for susceptibility tests performed in brain heart infusion broth with 10% fetal calf serum [24].

**Table 3:** MIC of six antimicrobial substances for one *Treponema *sp. isolate (V1) in nine subsequent susceptibility tests.

Days of incubation	MIC (μg/ml)					
	
	Tiamulin	Valnemulin	Tylosin	Aivlosin	Lincomycin	Doxycycline
8	0.5	0.125	≤0.5	≤0.25	>4	0.063
6^a^	0.5	0.125	≤0.5	≤0.25	>4	0.031
9^a^	1	0.125	≤0.5	≤0.25	>4	0.063
7	0.5	0.125	≤0.5	≤0.25	>4	0.063
10	0.5	0.063	≤0.5	≤0.25	>4	0.063
4^a^	0.5	0.063	≤0.5	≤0.25	>4	0.063
7^a^	0.5	0.125	≤0.5	≤0.25	>4	0.063
7	0.5	0.125	≤0.5	≤0.25	>4	0.031
11	0.5	0.063	≤0.5	≤0.25	>4	0.063
5	0.5	0.125	≤0.5	≤0.25	>4	0.063
9	0.5	0.125	≤0.5	≤0.25	>4	0.063

^a ^The same test was read twice with three days interval.

**Table 4:** MIC of six antimicrobial substances for seven *Treponema *sp. isolates.

Isolate	No. of tests performed	MIC (μg/ml)					
		
		Tiamulin	Valnemulin	Tylosin	Aivlosin	Lincomycin	Doxycycline
V1	9	0.5–1	0.063–0.125	≤0.5	≤0.25	>4	0.031–0.063
T 413	5	0.5–1	0.125	≤0.5	≤0.25	≥4	0.063
T 551	4	0.25–0.5	0.063–0.125	≤0.5	≤0.25	>4	0.031–0.125
T 551B	3	0.5	0.125–0.25	≤0.5	≤0.25	>4	0.063
T 603	2	0.5–1	0.125–0.25	≤0.5	≤0.25	>4	0.125
T 657	3	0.5	0.063–0.125	≤0.5	≤0.25	>4	0.125–0.25
T 2378	5	0.25–0.5	0.125	≤0.5	≤0.25	>4	0.031–0.063

### API-ZYM

The strength of the colour change in the API strips was read visually and judged on a scale 0–5 as suggested by the manufacturer. In Table 5 the reactions are presented as strong (S) 4–5; weak (W) 1–3 and negative 0. Isolate V1 was tested in both isotonic saline and PBS and incubated both aerobically and anaerobically. There was no difference caused by incubation atmosphere but the tests in PBS gave a positive reaction for α-fucosidase in both atmospheres that was not recorded in the isotonic saline tests. The results presented in Table 5 are all from tests performed in isotonic saline. All isolates had a similar enzymatic profile but T 551B differed through a weak positive reaction for leucine arylamidase. The results for the control strain *Pseudomonas aeruginosa *(ATCC 27853) were in agreement with the results given by the manufacturer except for one additional weak reaction (naphtol-AS-BI-phosphohydrolase).

**Table 5:** Enzymatic profile for seven *Treponema *sp. isolates determined by the API-ZYM system.

Isolate	Enzyme activity^a^																		
	
	1	2	3	4	5	6	7	8	9	10	11	12	13	14	15	16	17	18	19
V1	S	W	W	-	-	-	-	-	-	S	S	-	S	W	-	-	S	-	-
T 413	S	W	W	-	-	-	-	-	-	S	S	-	S	S	-	-	S	-	-
T 551	S	W	W	-	-	-	-	-	-	S	S	-	S	W	-	-	S	-	-
T 551B	S	W	W	-	W	-	-	-	-	S	W	-	S	W	-	-	S	-	-
T 603	S	W	W	-	-	-	-	-	-	S	W	-	S	W	-	-	S	-	-
T 657	S	W	W	-	-	-	-	-	-	S	W	-	S	W	-	-	S	-	-
T 2378	S	W	W	-	-	-	-	-	-	S	W	-	S	W	-	-	S	-	-

1 alkaline phosphatase; 2 C_4 _esterase; 3 C_8 _esterase lipase; 4 C_14 _lipase; 5 leucine arylamidase; 6 valine arylamidase; 7 cystine arylamidase; 8 trypsin; 9 α-chymotrypsin; 10 acid phosphatase; 11 naphtholphosphohydrolase; 12 α-galactosidase; 13 β-galactosidase; 14 β-glucuronidase; 15 α-glucosidase; 16 β-glucosidase; 17 N-acetyl-β-glucosaminidase; 18 α-mannosidase; 19 α-fucosidase.

^a ^S, strong; W, weak; -, negative.

### Pulsed-field gel electrophoresis

**Figure 1:**
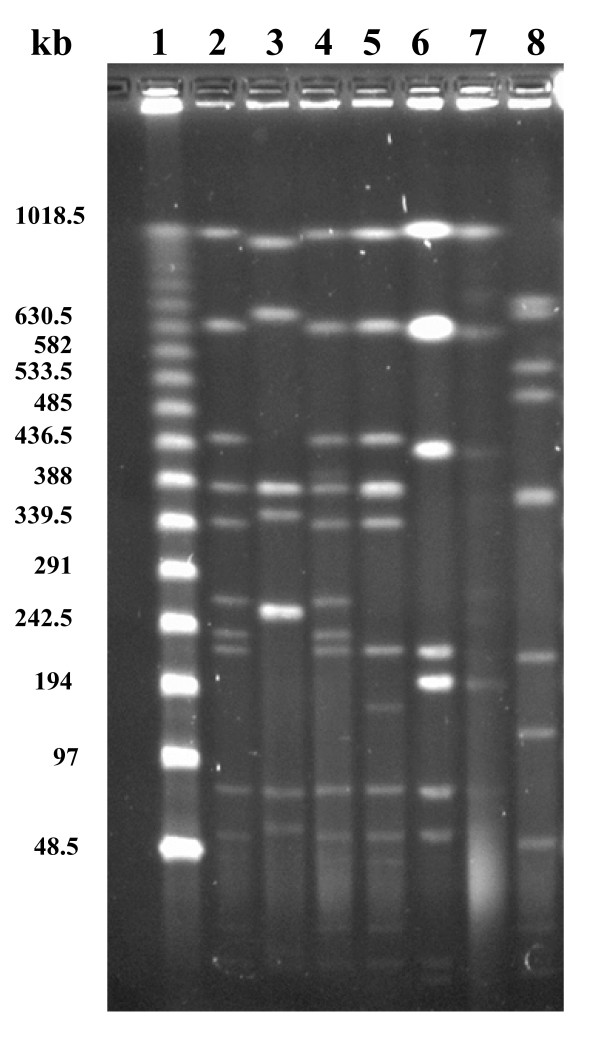
**Pulsed-field gel electrophoresis patterns for seven *Treponema *sp. isolates digested with *Xba*I.** Lane 1, Lambda Ladder PFG Marker (New England BioLabs); lane 2, V1; lane 3, T 413; lane 4, T 551; lane 5, T551B; lane 6, T 603; lane 7, T 657; lane 8, T 2378.

The PFGE banding pattern for the seven isolates are presented in Figure 1. Isolate V1 and T 551 had identical patterns but the others differed to various degrees. Cleavage of DNA from isolate T 657 generated weak bands and a smear at the bottom of the gel. Gel plugs with different cell densities from three different cultures and occasions were made for isolate T 657 without obtaining a distinct banding pattern.

### RAPD

**Figure 2:**
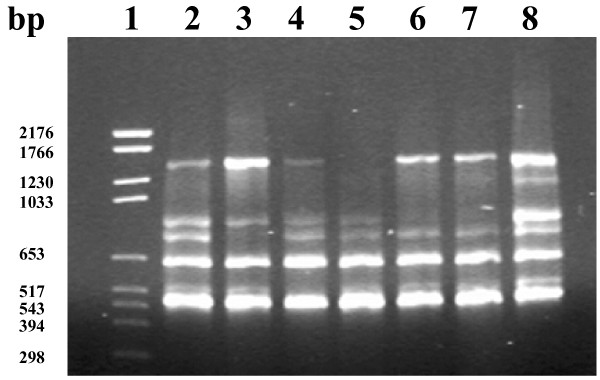
**Random amplified polymorphic DNA banding patterns for seven *Treponema *sp. isolates amplified with Primer 4, Ready-To-Go™ RAPD kit (GE Healthcare). **Lane 1, DNA Molecular Weight Marker VI (Roche Applied Science); lane 2, V1; lane 3, T 413; lane 4, T 551; lane 5, T551B; lane 6, T 603; lane 7, T 657; lane 8, T 2378.

The products from a Primer 4 RAPD reaction are shown in Figure 2. Reactions with isolate V1 and T 551 gave similar bands but some of the T 551 bands were weaker. The patterns of isolates T 603 and T 657 were also very similar.

## Discussion

To effectively treat DD, take preventive measures and start control programmes the cause/s of the disease needs to be identified and characterized. We describe seven isolates of a *Treponema *sp. from cattle with digital dermatitis, most closely related to *T. phagedenis*. The spirochete isolates from DD described to date originate from the USA (Iowa and California), the UK and Germany [8,11,14-16]. They represent three yet unnamed phylotypes and *T. brennaborense *and two thirds of the isolates belong to the *T. phagedenis*-like phylotype. However, phylotypes that have not been cultured have been identified through FISH and cloning of 16S rRNA genes from tissue samples [9,17,18]. A recent study, in which different combinations of *Treponema *phylotypes in biopsies from 56 dairy cows were analysed by FISH, showed that the *T. phagedenis*-like phylotype was the most prevalent and could be detected in 100% of the samples [18].

Compared to the most recent sequence deposited for *T. phagedenis *CIP62.29 [GenBank: EF645248] the 16S rRNA gene sequences from the isolates in this study have only one single nucleotide difference and with exception for T 603 and T 657 they are identical to sequences from DD isolates from Iowa, California and the UK [11,16]. In the ribosomal DNA intergenic spacer region study by Stamm et al. two operons for the ribosomal genes were identified in the *T. phagedenis*-like isolates [19]. In T 603 and T 657 three polymorphisms, which all had two nucleotides in 50% representation, also indicate that two operons are present. The polymorphisms were identical between the two isolates.

Antimicrobial susceptibility tests are essential to develop treatment regimens tailored to a specific etiologic agent. To be able to compare results the tests need to be standardized and for extremely fastidious bacteria such as *Treponema *spp. this is difficult. A broth dilution method developed for *Brachyspira *spp. (anaerobic spirochetes causing intestinal diseases) was modified and assessed. While it was not possible to make viable counts to standardize the inoculum density, fully grown cultures were approximated not to exceed 10^8 ^cells/ml which would give an inoculum density of somewhat less than 10^6 ^cells/ml. Even though the incubation time to obtain visible growth in the wells varied the MICs were unexpectedly stabile. Besides the long incubation the content of serum in the broth (25%) could also interfere with the effect of many antibiotics but the results for both control organisms tested were either within the recommended control ranges or one dilution step above. Repeated tests of isolate V1 did not vary more than one twofold dilution step (Table 3) which is also very stable considering that the acceptable variation of the method is plus or minus one twofold dilution. Taken all of this together we consider the antimicrobial susceptibility results in this study as reliable.

Except for lincomycin, for which the panel range was exceeded in most tests, the isolates in this study were susceptible to all antimicrobial substances tested. However, for all isolates at the time when the results were read, a decline in growth was observed in the well with the highest concentration of lincomycin. This observation, together with a lincomycin MIC of 4 μg/ml for one isolate indicates an MIC of 4–8 μg/ml for the remaining isolates. Tylosin (a macrolide) and lincomycin (a lincosamide) have overlapping binding sites on the ribosome and the MICs often follow each other. Compared to wild type *Brachyspira hyodysenteriae *[24] the lincomycin MIC for the *T. phagedenis*-like isolates is high whereas the tylosin MIC is low. The higher lincomycin MIC in the *Treponema *isolates could be explained by structural differences in 23S ribosomal RNA or ribosomal proteins. To assess if the *T. phagedenis*-like isolates in this study represents the wild type or if the binding site is altered, a larger number of isolates needs to be tested. Considering that DD has not been present long in Sweden and no footbaths with antibiotics are used, the high susceptibility is not surprising.

The enzyme activity was similar to what has been reported for other *T. phagedenis *like isolates [8,16]. In some earlier publications API-ZYM tests have been performed in PBS. As this is not recommended by the manufacturer we tested both PBS and isotonic saline for isolate V1 and found that the PBS test gave one additional positive reaction (α-fucosidase) compared to the non buffered isotonic saline. In the study performed by Evans et al. all *T. phagedenis*-like isolates were positive in the α-fucosidase reaction [16]. The subjective reading and different test conditions make interlaboratory results difficult to compare.

The PFGE protocol was modified from a protocol originally developed for *Campylobacter *spp. [23]. The density of the suspension of the treponemes for the gel plugs had to be approximately doubled compared to the original protocol. The protocol was not suitable for isolate T 657 despite that different cell and enzyme concentrations were tested. The DNA of this isolate seems to degrade, resulting in a smear at the bottom of the gel (Figure 1). The other methods applied on T 657 did not cause any problems.

The RAPD results showed a similar relationship between the isolates as found with PFGE. It should be born in mind that this method is susceptible to small changes and that only isolates within a single run can be compared with each other. However compared to PFGE the method is easier, cheaper and faster. For the seven isolates in this study Primer 4 in the kit gave a result that supported the PFGE findings, however this observation needs to be confirmed for a larger number of isolates.

A challenge study was performed with a culture of isolate V1 (unpublished, approved by the Ethical Committee on Animal Experiments, Gothenburg, Sweden). Claws of three dairy cattle were inoculated and the bandages were covered with rubber boots. During the early stage of the infection protocol, one cow (551), which came from a separate herd, was discovered to have interdigital DD. Despite this the protocol was carried out to completion in all cows. Isolate T 551 and T 551B included in this study are from this cow (Table 1). Both the PFGE and RAPD results indicate that V1 and T 551 are identical and if so, V1 was colonising cow 551 for at least 25 days. The isolate T 551B had unique PFGE and RAPD patterns and most probably originates from the herd of which cow 551 was a member. No typical lesions of DD were reproduced in any of the animals in the challenge trial.

Isolates T 603 and T 657 are from the same herd that is geographically remote from the other herds in the study. They have a similar PFGE pattern (one band that differs as interpreted after repeated tests, data not shown, see the results section for the problems with T 657) and identical RAPD results. They also have the polymorphisms in the 16S rRNA operons in common. This marker and the results for the fingerprinting methods, PFGE and RAPD, are in concordance with what could be expected, indicating that the two methods could be used to trace strains of this phylotype.

The pathogenic potential of *Treponema *spp. found in DD lesions needs to be studied both through identification of virulence traits and ultimately through fulfilment of Koch's postulate. To use mixes of different *Treponema *phylotypes could possibly be the solution to reproduce the disease.

## Conclusion

The results from this study show that Swedish cattle with DD are colonized with a *Treponema *sp. that also has been cultured from samples from cattle in the USA and the UK and is closely related to *T. phagedenis*. While very similar, the isolates studied are possible to differentiate through PFGE and RAPD indicating that these methods are suitable for subtyping of this phylotype. All isolates were susceptible to the antimicrobial agents in the panel used.

## Competing interests

The authors declare that they have no competing interests.

## Authors' contributions

MP carried out most of the laboratory analyses, the interpretation of the results and the manuscript preparation. LLF carried out the RAPD analysis and contributed to the evaluation of the results. HH and MP adapted the PFGE protocol for the phylotype studied. CB, HH, and KEJ participated in planning of the investigation and critically reviewed the manuscript. All authors read and approved the final manuscript.
